# Association Study of Estrogen Receptor Alpha Gene Polymorphisms with Spontaneous Abortion: Is This a Possible Reason for Unexplained Spontaneous Abortion?

**DOI:** 10.1155/2013/256470

**Published:** 2013-10-20

**Authors:** Negin Anousha, Arash Hossein-Nezhad, Firouzeh Biramijamal, Ali Rahmani, Zhila Maghbooli, Elahe Aghababaei, Shahram Nemati

**Affiliations:** ^1^Department of Genetics, Ahar Branch, Islamic Azad University, Ahar 54511, Iran; ^2^Department of Medicine, Section of Endocrinology, Nutrition, and Diabetes, Vitamin D, Skin and Bone Research Laboratory, Boston University Medical Center, Boston, MA 02118, USA; ^3^Endocrinology and Metabolism Research Institute, Tehran University of Medical Sciences, Tehran 14114, Iran; ^4^Department of Medical Genetics, National Institute of Genetic Engineering and Biotechnology, Tehran 14178, Iran

## Abstract

Estrogen plays a crucial role in fetal and placental development through estrogen receptors. Association of estrogen receptor alpha gene (*ESR1*) polymorphisms with spontaneous abortion has been shown in some studies. Our main goal was to study the potential association of spontaneous abortion with the *ESR1* gene variations (*PvuII* and *XbaI*) in fetal tissue. Totally, 161 samples were recruited including 80 samples of formalin-fixed paraffin-embedded fetal tissue from spontaneous abortion and 81 samples of normal term placental tissue. The restriction fragment length polymorphism (RFLP) method was performed for genotyping the rs2234693 (A/G *XbaI*) and rs9340799 (T/C *PvuII*) single nucleotide polymorphisms located in intron 1 of *ESR1*. The results have been confirmed by DNA sequencing analysis. The different genotypes distribution was detected in two study groups. Haplotype analysis indicated that *ppxx* is protective genotype against spontaneous abortion (*P* = 0.01). In conclusion, the potential role of *ESR1* genetic variation in spontaneous abortion might be valuable in high-risk subjects, and that needs to be confirmed with future studies.

## 1. Introduction

Estrogen receptors (ER) are group of proteins classified as nuclear receptors and member of ligand-activated transcription factors. Estrogen actions on target tissues are mediated by the ER [1]. Estrogen is a major endocrine hormone, playing a crucial role throughout the entire pregnancy such as fetal development, uteroplacental blood flow, implantation, regulation of reproduction, and biosynthesis of progesterone [2, 3]. It has been reported that a huge amount of estrogen is produced by human placenta during pregnancy. Recent studies showed that estrogen seemed to have a vital role in the development of placental villous blood vessel. A noticeable increase in biosynthesis of placental estrogen was observed in the early stages of pregnancy when the placental vascular network starts to develop [4]. The autocrine role of placenta in estrogen production is critical for trophoblast cells differentiation [5]. Blockage of estrogen receptor as a consequence of tamoxifen, given orally to female bonnet monkeys, led to pregnancy inhibition through postovulatory period [6]. ER*α* and ER*β*, as two types of ER, are encoded by ESR1 and ESR2 genes located on chromosome 6 and chromosome 14, respectively [7, 8]. ER*α* is more abundant and exists in all human reproductive tissues [2]. Expression of ER*α*, but not ER*β*, has been observed in cultured human trophoblast cells before and after differentiation [9]. ER*α*-knockout female mice were anovulatory and infertile. The uteri of these mice did not respond to estrogen [10].

Spontaneous abortion (SA) is an unintentional end of pregnancy before the stage that the fetus is able to survive independently [11]. SA is one of the most common complications of pregnancy, particularly in the first trimester, which occurs in approximately 15%–20% of clinically recognized pregnancies [12, 13]. The pathogenesis of SA is multifactorial, and approximately 40%–50% of recurrent spontaneous abortions remain idiopathic [11]. Genetic variations might be noticeable in idiopathic SA, and the association of several genetic variations with SA has been reported [14]. The *ESR1* was one of the best candidate genes in SA association studies, and its polymorphisms were involved in SA [3, 15]. Furthermore, the role of numerous polymorphic variants in the fertility candidate genes including ESR1 is consistent with this hypothesis [16, 17].

The *ESR1* has been reported as an extremely polymorphic gene, such that studies determined more than 2200 SNPs of this gene [18]. The rs2234693 (T>C: *PvuII*) and rs9340799 (A>G: *XbaI*) located in the intron 1 have been identified as the most common and widely studied SNPs in related investigations [18, 19]. These common *ESR1 *polymorphisms have been reported in relation with SA [3], infertility [20], successful IVF (in vitro fertilization) [18, 21], and preeclampsia [22]. Although these intronic variations (*PvuII* and *XbaI)* were not involved in protein changing, they have been suggested as genetic markers for some *ESR1*-related disorders due to a linkage with other functional sequences affecting ESR1 function or expression [21, 22].

In spite of the growing body of evidence regarding the potential role of *ESR1* genetic variations in SA, the association of these polymorphisms (*PvuII* and *XbaI*) has not been investigated in human fetal genome.

In this study, the possible association of *PvuII* and *XbaI* polymorphisms of the *ESR1* with spontaneous abortion was evaluated in spontaneously aborted fetus tissue in comparison with placental tissue of healthy term newborns.

## 2. Materials and Methods

### 2.1. Subjects

The study protocol was approved by ethics committee of EMRI (Endocrinology and Metabolism Research Institute of Tehran University of Medical Sciences). Informed written consent was obtained from all participants. Eighty samples of formalin-fixed paraffin-embedded fetal tissue from spontaneous abortion diagnosed previously were included as a case group. These samples were collected from Emam-Sajad and Ghiasi Hospitals of Tehran cooperating with EMRI.

The samples of this group were selected by the inclusion criteria of containing fetal tissue from spontaneous abortion in the first trimester of gestation during natural pregnancies and of unknown etiology confirmed by a gynecologist and a pathologist. All of the case samples were karyotyped, and the samples with abnormal karyotype and infections including toxoplasmosis, rubella, cytomegalovirus, HIV, group B streptococci, chlamydia trachomatis, and hepatitis virus B and C were excluded. The case samples were compared with a control population consisted of 81 samples of normal placental tissue. The healthy controls included in this study were all from fetal portion of term placental tissue and were collected immediately after term delivery.

### 2.2. Genomic DNA Analysis

Genomic DNA was extracted from formalin-fixed paraffin-embedded fetal tissue and normal term placental tissue (fetal portion) samples using i-genomic CTB DNA Extraction Mini Kit according to the protocol (iNtRON Biotechnology, Inc., Korea). To determine the genotypic pattern of *PvuII* (T > C, rs2234693) and *XbaI* (A > G, rs9340799) polymorphisms in intron 1 of ESR1 gene, we used polymerase chain reaction followed by restriction fragment length polymorphism method (PCR-RFLP). A PCR fragment of 346 bp consisting of the both base pair changes by the primers (forward primer: 5′-GATATCCAGGGTTATGTGGCA-3′ and reverse primer: 5′-AGGTGTTGCCTATTATATTAACCTTGA-3′) was amplified [3]. The total volume of PCR reaction was 20 *μ*L containing 50 ng genomic DNA, 10 pM of primers, 0.2 mM dNTP, 2 mM MgCl_2_, 2 *μ*L of 10x buffer, and 1 U of *Taq *DNA polymerase (Fermentas, Vilnius, Lithuania). PCR conditions were as follows: initial denaturation at 94°C for 5 min, 35 cycles with denaturation at 94°C for 45 sec, annealing at 53°C for 45 sec, and extension at 72°C for 45 sec followed by 1 cycle of a final extension at 72°C for 7 min. After performing the PCR, the amplification product of 346 bp was digested overnight at 37°C with *PvuII* and *XbaI* restriction enzymes (TaKaRa, Otsu, Japan). The digested products were detected on 3% agarose gel electrophoresis.

For rs9340799 SNP after the digestion of 346 bp amplified product, two fragments of 148 and 198 bp were observed in the presence of *XbaI* restriction site in which *A* allele is present. For rs2234693 SNP, in the presence of *T* allele due to the presence of *PvuII* restriction site, two fragments of 103 and 243 bp were identified from a digested 346 bp amplified product. The presence of restriction site was indicated by “*p* or *x*” allele, while the absence was shown by “*P* or *X*” allele for *PvuII* and *XbaI* polymorphisms, respectively.

Fifteen percent of the PCR samples were sequenced to confirm the PCR-RFLP results using the ABI PRISM 3730 automated sequencer (Applied Biosystems, Foster City, Calif).

### 2.3. Statistical Analysis

Genotype frequencies were compared in cases and control groups. The SPSS version 16 software was used for all of the statistical analysis. Quantitative variables were evaluated by the student's *t*-test, while the *χ* ^2^ test was used for analyzing the qualitative variables. The agreement with the Hardy-Weinberg expectations for the genotype distributions was also confirmed by *χ* ^2^ test. A *P* value ≤ 0.05 was considered to be statistically significant.

## 3. Result

Totally, 161 tissue samples were recruited in this study. These samples were provided from 80 spontaneously aborted fetuses that were kept as formalin-fixed paraffin-embedded tissue as spontaneous abortion (SA) group, and 81 normal term placental tissue as control group.

The mean of maternal age in healthy and SA groups was 27.7 ± 5.7 and 28.08 ± 5.4 years, respectively (*P* = 0.7). The genotype distributions of *PvuII* and *XbaI* polymorphisms in studied population were in the Hardy-Weinberg equilibrium.

**Figure 1:**
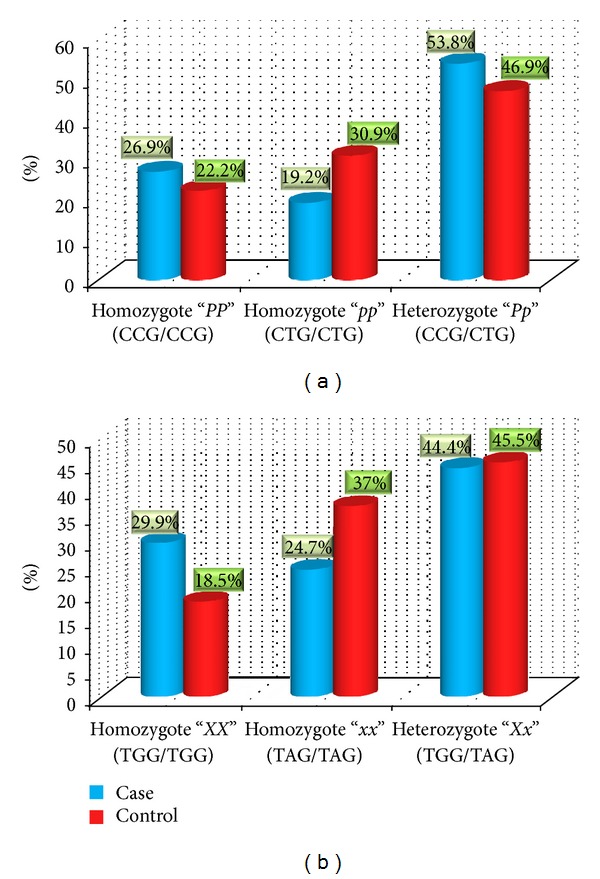
(a) The distribution of *PvuII* genotypes among spontaneous abortion group and control groups (*P* = 0.2). (b) The distribution of *XbaI* genotypes among spontaneous abortion group and control groups (*P* = 0.1). The horizontal axis shows the three genotypes of each SNP, and the vertical axis shows the genotype frequencies in the spontaneous abortion and control groups.

The frequencies of *PvuII* genotypes in healthy controls were 18 (22.2%), 25 (30.9%), and 38 (46.9%) for *PP, pp*, and *PpI*, respectively. The frequencies of *PP, pp*, and *Pp* in SA group were 21 (26.9%), 15 (19.2%), and 42 (53.8%), respectively. The frequencies of *XX, xx*, and *Xx* genotypes were 15 (18.5%), 30 (37.0%), and 36 (45.5%) in healthy controls, as well as 23 (29.9%), 19 (24.7%), and 36 (44.4%) in the SA group, respectively (Figure 1).

The frequency of *pp* genotype of the *PvuII* and *xx* genotype of *XbaI* were lower in SA group compared to those of healthy controls (*pp* versus *PP* + *Pp* and *xx* versus *XX* + *Xx*; *P* value = 0.09). However, there were no statistically significant differences between SA and healthy groups in genotype distribution of ESR1 *PvuII* and *XbaI *separately. In haplotype analysis *ppxx* genotype was significantly more frequent in healthy fetus in comparison to SA group (*P* = 0.01, odds ratio = 2.530, 95% CI: 1.167–5.485) (Table 1).

As shown in Table 2, a significant correlation was found between the ESR1 *XbaI* and *PvuII* polymorphisms (*PP* with *XX* and *pp* with *xx* genotypes) (*P* < 0.01), reflecting the known linkage disequilibrium between the two SNPs [23].

## 4. Discussion

Estrogen plays a pivotal role during pregnancy. Studies reported that a considerable amount of estrogen is raised by the human placenta throughout pregnancy. It is completely known that estrogen produced by placenta is involved in the physiological procedures which are crucial for fetal growth and development during both extrauterine and intrauterine periods of life [4]. Since estrogen effects are modulated through estrogen receptors, variations in the *ESR* could have an important influence on its related procedures that provide pregnancy maintenance [15].

Some contradicting results of the role of *ESR1* polymorphisms in spontaneous abortion or infertility were reported. In this study, for the first time, we investigated a possible association between *ESR1* variants and spontaneous abortion in the fetal genome.

Several studies have reported strong linkage disequilibrium between *PvuII* and *XbaI* polymorphisms. These polymorphisms are located in the first intron of *ESR1*, and the linkage disequilibrium might be as a result of approximately 50 bp distance between them. Such that allele *p* (*PvuII* positive restriction site) is associated with *x* allele (*XbaI* positive restriction site) as well as allele *P* (*PvuII* negative restriction site) being linked with *X* allele (*XbaI* negative restriction site) [2, 22, 23]. Interestingly, in our study we found a significant correlation of *pp* genotype of *PvuII* polymorphism with *xx* genotype of *XbaI* and *PP* genotype with *XX* as well (*P* = 0.000).

Also, our findings demonstrated a lower frequency of *pp* and *xx* genotypes of *PvuII* and *XbaI* polymorphisms in the SA group compared with that in healthy controls.

A genetic haplotype is identified as a combination of sets of alleles on the same chromosomal segment that tend to be transmitted as a block [24]. When we combined *PvuII* and *XbaI* genotypes into haplotypes, a statistically significant difference between control and SA groups for *ppxx* genotype combination with the higher frequency of *ppxx *in the healthy controls was observed (*P* = 0.01), verifying the protective role of *ppxx* genotype against spontaneous abortion. The consistent results were observed in a study of the *PvuII *and* XbaI* gene polymorphisms on a sample of Italian women in which Corbo et al. demonstrated a significantly lower number of abortions among *pp* homozygote women compared with the carriers of *PP* or *Pp* genotypes. Furthermore, a significant association of *ppxx* genotype with lower number of abortions among the Italian women was reported. Simultaneously, in a sample of African-Ecuadorian women, it had been indicated that women carrying *pp* and *ppxx* genotypes were correlated with higher number of children [2]. Also, Corbo et al. demonstrated that various reproductive behaviors and environment may affect the impact of *ESR1* genotypes on fertility [2, 17]. In another study among premature infants, Derzbach et al. showed that any homozygotes for *PvuII* polymorphism, including *PP* or *pp*, have a higher risk of at least one of the most common complications in perinatal period [25].

M'Rabet et al. demonstrated a potential role of *PvuII* allelic variants for infertility prediction among women at risk of premature ovarian aging with a higher frequency of the *PP* genotype of *PvuII* polymorphism [26].

Pineda et al. found that the maternal *TA* haplotype (or *px*) of *PvuII* and *XbaI* polymorphisms were associated with the increased risk of spontaneous abortion in women with miscarriages [3].

Other study in relation to unexplained female infertility detected the *P* allele of *PvuII* polymorphism as a risk factor for females' idiopathic infertility, whereas *X* allele of *XbaI* had been reported to play a protective role regarding this condition [27].

In other studies, enhanced quality and number of follicles, mature oocytes, and fertilization rate among patients undergoing IVF were associated with PvuII CC (PP) genotype of ESR1 [18, 20, 21, 28].

Silva et al. revealed an association of *xx* and *ppxx* genotypes of *PvuII* and *XbaI* polymorphisms with spontaneous abortion in a population of postmenopausal women [29].

Since ethnic/race specific associations of ESR1 gene have been reported [24], contradiction among different investigations could be attributed to variations in ethnic groups. Indeed, the specific influence of gene polymorphisms and environmental factors could be different in various populations.

Several mechanisms could be explained for phenotypic effects of an intronic polymorphism [22]. Weickert et al. indicated that decreased levels of *ESR1 *mRNA were associated with *PP* genotype of *PvuII* polymorphism in postmortem brain tissue of schizophrenics. Reduced stability of *ESR1* mRNA was found to be a result of *P* allele incidence [30]. It is demonstrated that *ESR1* gene polymorphisms can alter the function of estrogen receptor, but the exact biological mechanism remains unclear (or unknown) yet [24].

It has been reported that the increased gene enhancer activity might alter transcription levels of the gene, but it remains disputed so far that how an enhancer exactly exerts its effect [31].

In vitro studies have revealed a different activity of enhancer, although not significantly, between *ESR1* haplotypes. Interestingly, the maximum enhancer activity was found to be associated with *x* allele and *ppxx* genotype [32]. Thereby, regulation of *ESR1* expression could be biased by *ESR1* genetic variants [2]. Hence, by modifying *ESR1* function, biological actions of estrogen as one of the most influential and prominent hormones for fetal-placental development throughout pregnancy are placed under the influence [2, 4].

Since there has not been sufficient research in relation to effects of fetal genetic variations of *ESR1 *on reproduction efficiency and risk of spontaneous abortion, more prospective studies with larger sample size in different populations are recommended. Also, the evaluating of *ESR1* expression in relation with *PvuII* and *XbaI* can be helpful to clear the functional role of these variations. This was a limitation of the current study that due to using formalin-fixed paraffin-embedded fetal tissue as sources of SA group, we could not asses this evaluation. Therefore, further investigations on *ESR1* expression level influenced by the *PvuII* and *XbaI* gene polymorphisms in spontaneously aborted samples are required as well.

In conclusion, our results indicated a meaningful association of *ppxx* genotype with the decreased risk of spontaneous abortion. According to the profound effects of estrogen on fetal growth and development, we hypothesize that reduced estrogen activity due to the variations in the *ESR1* of the fetal genome could affect fetal and placental development and stability during pregnancy. Accordingly, our study suggests *ESR1* as a candidate gene for spontaneous abortion. However, further investigations need to confirm the impact of single nucleotide polymorphisms on spontaneous abortions among high-risk subjects.

## Figures and Tables

**Table 1:** Haplotype frequencies of *PvuII* and *XbaI* polymorphisms in healthy and spontaneous abortion groups.

Group	Genotypes, *n* (%)	
	*ppxx*	Other combined genotypes
Spontaneous abortion	12 (15.0%)	68 (85.0%)
Healthy	25 (30.9%)*	56 (69.1%)

*P = 0.01 (*P* value, two-sided, from *χ* ^2^ test).

**Table 2:** The correlation of *PvuII* and *XbaI* polymorphisms in the population.

	PVUII*		
	*PP*	*pp*	*Pp*
XbaI*			
*XX*% (*N*)	86.8 (33)*	2.6 (1)	10.5 (4)
*xx*% (*N*)	2 (1)	75.5 (37)*	22.4 (11)
*Xx*% (*N*)	7 (5)	1.4 (1)	91.5 (65)

*P = 0.000.
